# Systematic review of peri-implant conditions and aesthetic outcomes of customized versus conventional healing abutments

**DOI:** 10.1186/s40729-024-00581-8

**Published:** 2024-12-11

**Authors:** Miriam Ruhstorfer, Jan-Frederik Güth, Michael Stimmelmayr, Lukas Waltenberger, Oliver Schubert, Tobias Graf

**Affiliations:** 1 Department of Prosthodontics, Center for Dentistry and Oral Medicine (Carolinum), Goethe University, Frankfurt Am Main, Germany; 2 Department of Prosthetic Dentistry, University Hospital, LMU Munich, Munich, Germany; 3 Department of Prosthodontics and Biomaterials, Center for Implantology, Uniklinik RWTH Aachen, Aachen, Germany

**Keywords:** CAD/CAM, Dental implant, Emergence profile, Healing abutment, Pain development, PROMs, Soft tissue healing

## Abstract

**Purpose:**

Customized healing abutments are utilized to enhance aesthetics and peri-implant soft and hard tissue health, and play a crucial role in the implant-prosthetic workflow. This systematic review was performed to assess and compare the clinical outcomes of customized healing abutments with conventional ones.

**Methods:**

The review was registered with PROSPERO (ID: CRD42024532449) and followed the PRISMA-guidelines. The PICO-question addressed was: “In patients with dental implants, do customized healing abutments result in beneficial peri-implant conditions compared with conventional healing abutments?” Clinical trials involving immediate and late implant placement that compared different healing abutments based on quantifiable outcomes were included. The “PubMed”, “PubMed Central”, “Cochrane Library”, and “Web of Science databases” were screened for eligible studies until 4/20/2024.

**Results:**

Of 1,396 titles retrieved, 5 studies met the inclusion criteria and were analyzed. The included studies showed a low risk of bias as assessed by the RoB2 tool and Joanna Briggs Institute Critical Assessment questionnaire. Compared to conventional healing abutments, customized abutments were associated with a trend toward better clinical outcomes in peri-implant soft and hard tissue, as well as aesthetics. Several results within the cohorts using customized healing abutments showed significantly improved values in soft and hard tissue results as well as aesthetic parameters. Importantly, none of the included studies reported biological or aesthetic disadvantages associated with the use of customized healing abutments.

**Conclusions:**

Customized healing abutments maintain stability of peri-implant hard and soft tissue. However, further studies with larger sample sizes and longer follow-up periods are needed to validate these findings.

**Graphical Abstract:**

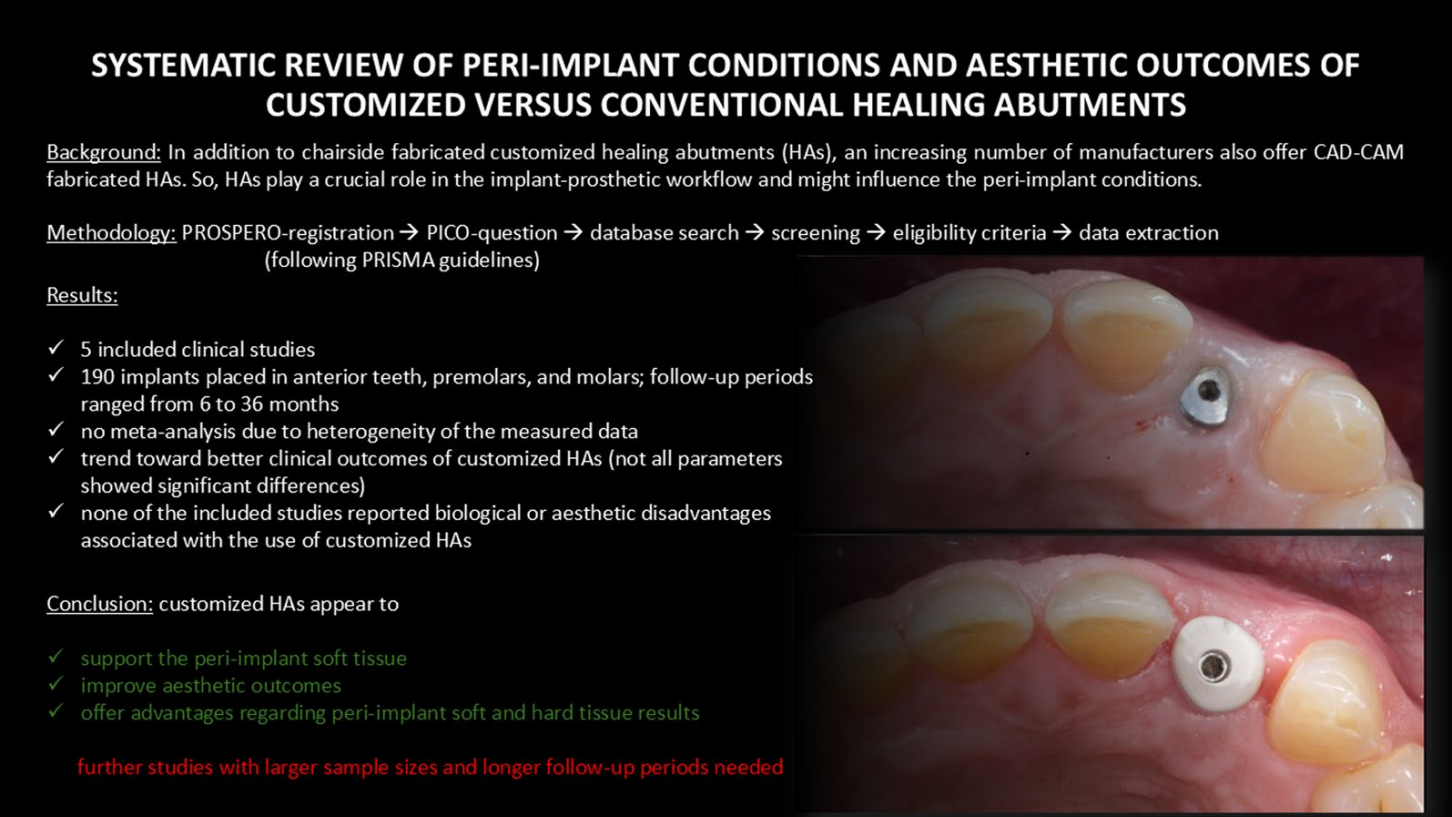

## Background

Since the 2000s, the rapid advancement of digital technology has introduced numerous new approaches, concepts, and workflows in diagnostics, planning, and treatment, particularly in implantology and implant prosthetics. These advancements aim to maximize predictability and efficiency while minimizing complications over time [1–4]. Consequently, the trend toward using implants and their prosthetic superstructures as standard treatment options continues to grow. For instance, the number of implant-supported dental prostheses in Germany tripled between 2005 and 2014, and patient demand is expected to remain strong in the future [5].

Patients often have high expectations for implant-supported prostheses. Considering the desire for improved aesthetic outcomes, adequate functional aspects, and cost-efficient treatment options, achieving good and stable long-term performance of prostheses is highly preferred [6–8].

Long-term stability hinges on effective soft tissue management following implant placement [9, 10]. Several strategies exist to shape and maintain peri-implant soft tissue during the healing phase. For immediate implants, prefabricated customized abutments or individualized stock or temporary abutments help maintain soft tissue and enable primary wound closure [11, 12]. Another approach is the one-abutment/one-time concept, which, when applied either during implant placement or at the time of uncovering the implant, tends to yield slightly better peri-implant biological outcomes [13–17]. Immediate restorations after implant placement are only feasible in selected cases. So, submerged or non-submerged healing processes combined with healing abutments are more commonly used.

Healing abutments are crucial in promoting the healing of peri-implant soft and hard tissue during the healing period, initiating soft tissue contouring, and protecting the implant site from plaque or debris during the initial post-surgical phase [18]. As such, they are pivotal tools in implantology and implant prosthetics.

In cases of late implant placement with submerged healing, conventional healing abutments are typically screwed in during the uncovering session as part of standard procedure [10, 19–21]. Conversely, immediate implant placement with non-submerged healing involves inserting healing abutments directly after implant placement, eliminating the need for a separate uncovering appointment [22].

Subsequently, the emergence profile is typically shaped by inserting the temporary or final prosthesis, often in several steps, under active compression of the peri-implant soft tissue [23]. This “secondary” soft tissue shaping demands a skilled team of clinicians and dental technicians because it presents significant biological challenges [24]. Moreover, the insertion of prostheses under tissue compression can be painful for patients [25, 26] and might lead to soft tissue recession.

Prefabricated round titanium healing abutments, available in various gingival heights, diameters, and configurations, are commonly used. Alternatively, (semi-)customized healing abutments can be employed to shape the peri-implant mucosa to a more natural profile of an emerging tooth [11, 18, 27–36]. This approach may avoid the time-consuming and uncomfortable “secondary” soft tissue shaping, though it also requires a well synchronized team of surgeons, dental technicians, and prosthodontists. Customized healing abutments can streamline the workflow but may also influence peri-implant soft tissue [24, 37].

The primary aim of this systematic review was to evaluate the available literature on the outcomes of customized healing abutments compared to conventional ones, focusing on stability of peri-implant soft- and hard tissue as well as aesthetic outcomes. Additionally, the review sought to determine which procedure results in better patient-reported outcome measures (PROMs) during treatment.

The null hypotheses were that customized healing abutments have no effect on the preservation of peri-implant (a) soft and (b) hard tissues, (c) aesthetic outcomes, or (d) PROMs compared with conventional healing abutments.

## Methods

### Study design and focused questions

This systematic review was registered on the National Institute for Health Research PROSPERO, the International Prospective Register of Systematic Reviews, (Prospero ID: CRD42024532449) on 17 April 2024 and follows the guidelines of the Preferred Reporting Items of Systematic Reviews and Meta-Analyses (PRISMA) [38]. The aim of this study was to evaluate clinical differences between customized and conventional healing abutments in patients with dental implants. The primary outcomes assessed and compared were quantifiable measurement values concerning changes/presence of peri-implant soft- and hard tissue, peri-implant tissue health status as well as aesthetic results. The secondary outcome examined was the PROMs associated with the clinical treatment workflows. Accordingly, the following PICO-question was developed: “In patients with dental implants, do customized healing abutments result in beneficial peri-implant conditions compared with conventional healing abutments?” (Table 1).

**Table 1:** Search strategy based on PICO question

PICO-Question	**In patients with dental implants, do customized healing abutments result in beneficial peri-implant conditions compared with conventional healing abutments?**		
Population (#1)	**P = Patients with inserted dental implants** • Dental implant* • Oral implant* • Endosseous implant* • Implant fixture* • “Dental Implants”[Mesh] • “Dental Implantation, Endosseous”[Mesh]		
Intervention (#2)	**I = Customized Healing Abutment**		
	• Sealing socket abutment • Seal socket abutment		
	• Customized • Custom • Individualized • Individual • Personalized	AND	• Healing abutment • Gingiva former • Healing cap • Healing screw
Comparison (#3)	**C = Conventional Healing Abutment**		
	• Conventional • Prefabricated • Transmucosal • Standard • Machined • Prefabricated • Titanium	AND	• Healing abutment • Gingiva former • Healing cap • Healing screw
Outcome (#4)	**O = Peri-implant conditions, aesthetic outcomes** • Objective parameters that evaluate peri-implant conditions, bone stability or signs of peri-implantitis or mucositis • Objective parameters that evaluate aesthetic outcomes		
Search combination	#1 AND (#2 OR #3)		

### Search strategy

A search strategy was developed based on the defined PICO question, which was executed as an electronic search in the PubMed, PubMed Central, Cochrane, and Web of Science databases. The search syntax was constructed using Medical Subject Headings (MeSH®) terms and various combinations of free-text words. Different terminologies are used in the literature for “customized healing abutments” and “conventional healing abutments”, which was taken into account by including synonymous terms in the search (Table 1). The publication period for eligible studies was set until 20 April 2024. Gray literature was excluded; however, the reviewers conducted a hand search, including references from the finally included articles.

The following is an example of the search path used in the PubMed database: “(dental implant* OR oral implant* OR endosseous implant* OR implant fixture* OR “Dental Implants”[Mesh] OR “Dental Implantation, Endosseous”[Mesh]) AND (Sealing socket abutment OR Seal socket abutment OR ((Customized OR Custom OR Individualized OR Individual OR Personalized) AND (healing abutment OR gingiva former OR healing cap OR healing screw)))”.

Clinical studies were included if they met the following criteria:

- Human studies.
- Patients with at least one customized healing abutment after immediate or late implant placement.
- Presence of a comparison group.
- Same healing method in both groups being compared (i.e., both cohorts underwent either submerged or non-submerged healing).
- Clinical study with a prospective or retrospective design.
- At least five patients per group.
- At least 6 months of follow-up.
- Articles published in English.
- Studies reporting on quantifiable peri-implant health, hard and/or soft tissue, or esthetic parameters around the inserted implant.

Studies that did not meet these inclusion criteria were excluded. Additional exclusion criteria were as follows:

- Systematic or narrative reviews.
- Case reports and technical reports.
- Comparison between submerged and non-submerged healing cohorts.
- Gray literature.
- Absence of quantifiable parameters.
- Insufficient information on defined outcome criteria.
- Multiple publications on the same patient population.
- Zygoma, pterygoid, and orthodontic implants.

### Study selection and data extraction

All articles were independently screened by title for relevance to the topic, and non-relevant articles were excluded (executed by TG, MR). Following this, the abstracts of the remaining articles were assessed for eligibility and relevance (executed by TG, MR). Full-text articles were then obtained based on abstract selection. In the final stage, the full-text articles were reviewed against the inclusion and exclusion criteria, focusing on the Materials and Methods, Results, and Discussion sections (executed by TG, MR). Any disagreements were discussed and resolved after each stage (executed by JFG, TG, MR). Studies were only included if all reviewing authors reached a consensus. No articles were excluded due to non-consensus.

Two reviewers (TG, MR) independently extracted data from each full-text article. Extracted data were compared and double-checked. For missing or unclear information, the corresponding authors of the respective studies were contacted via email.

The following data were summarized in a data extraction form: author(s), year of publication, study design, follow-up duration, study population, age of study population, timing of implant placement, implant positions, implant systems, method of implant placement (guided versus non-guided), gap-filling graft material, specification of healing abutment, inserted prosthetic restoration, implant/prosthetic survival/success rates, randomization process, primary and secondary outcome parameters, and conclusions.

### Quality assessment

The quality of the selected observational studies was evaluated according to the Strengthening the Reporting of Observational Studies in Epidemiology (STROBE) guidelines and double-checked (executed by TG, MR). All included studies were categorized based on their methodological design. Randomized clinical trials were assessed using the Revised Cochrane risk-of-bias tool for randomized trials (RoB2 tool) [39, 40], and cohort studies were evaluated using the Joanna Briggs Institute Critical Assessment questionnaires [41]. A risk-of-bias judgment was proposed based on standardized signaling questions, such as those concerning the randomization process, intended interventions, reported results, or missing outcome data.

### Data analysis

Due to heterogeneity of data, a meta-analysis was considered inappropriate. Thus, only qualitative analysis was conducted.

## Results

### Included studies

**Fig. 1:**
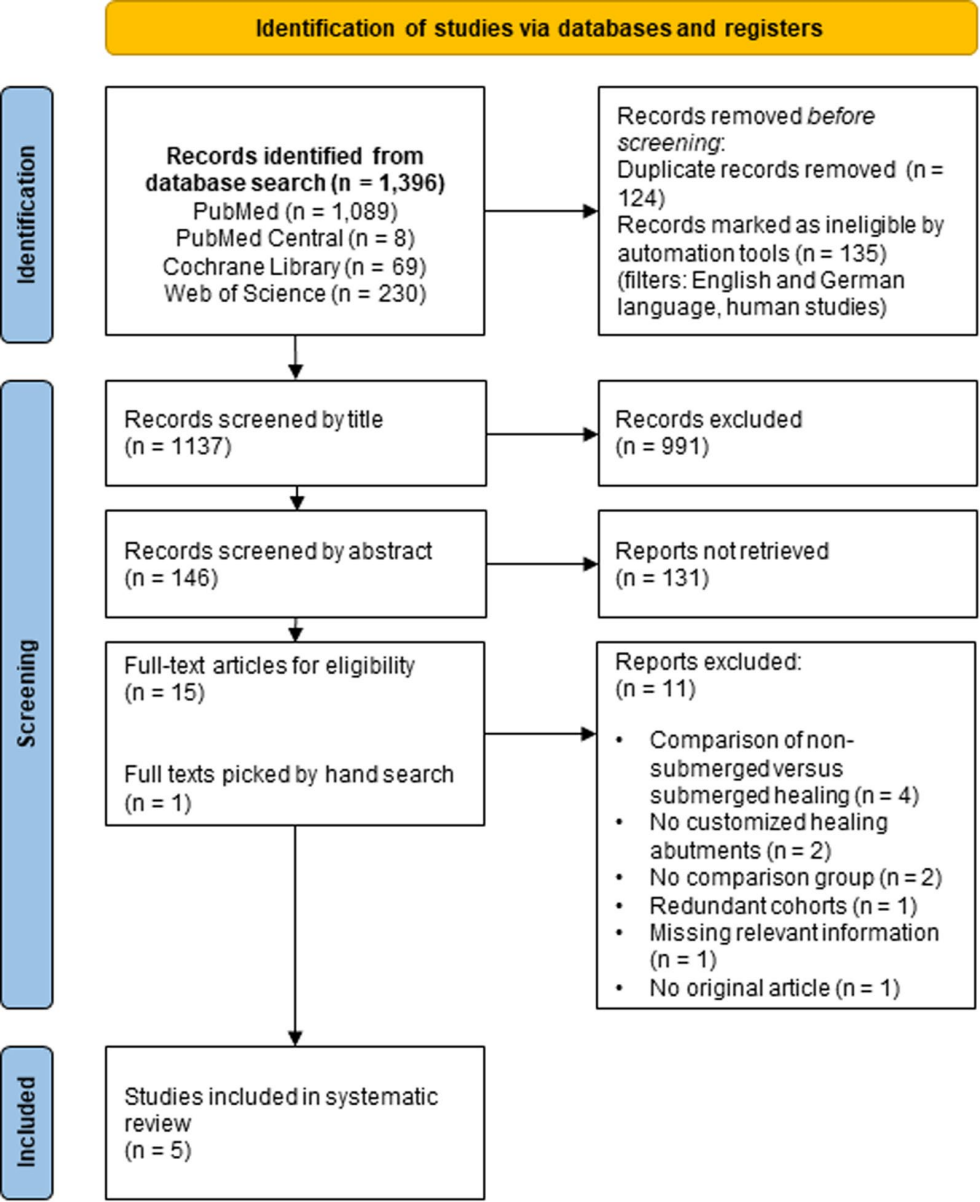
Study selection via traditional PRISMA flow diagram

In total, 1,396 titles were retrieved from the electronic search of the PubMed, PubMed Central, Cochrane Library, and Web of Science databases. These titles were screened for duplicates and ineligible studies using automation tools, resulting in the removal of 259 entries. Subsequently, 991 articles were excluded based on their titles. The remaining 146 articles were further screened based on their abstracts, leading to the exclusion of 131 additional articles. Ultimately, 15 articles, along with 1 additional article identified through a manual search, were selected for full-text review. This process culminated in the inclusion and analysis of five articles in the present review (Fig. 1, Table 2) [20, 26, 28, 42, 43].

**Table 2:** Overview of studies included and excluded after full-text review

Author(s)	Publication year	Incl	Excl	[if excluded] Reason for exclusion	Others
Abduo et al. [44]	2017		x	No comparison group	
Amato et al. [20]	2022	x			
Beretta et al. [25]	2019		x	Missing of relevant information (no feedback from authors after e-mail request)	
Borie et al. [45]	2022		x	No original article	
Borie et al. [46]	2020		x	No customized healing abutments	
Chokaree et al. [26]	2024	x			
Crespi et al. [29]	2023		x	No comparison group	
*Crespi *et al*.* [28]	*2023*	*x*			*Picked by hand search*
Crespi et al. [47]	2022		x	Redundant cohort (Cohort of present study was also included in *Crespi *et al*.* [29]. Information received from the corresponding author after e-mail request)	
Fernandes et al. [48]	2021		x	Comparison of non-submerged versus submerged healing	
Hu et al. [42]	2018	x			
Menchini Fabris et al. [30]	2023		x	Comparison of non-submerged versus submerged healing	
Menchini Fabris et al. [31]	2020		x	Comparison of non-submerged versus submerged healing	
Perez et al. [43]	2020	x			
Sanchez-Siles et al. [49]	2018		x	No customized healing abutments	
Wang et al. [32]	2021		x	Comparison of non-submerged versus submerged healing	

A detailed summary of the included studies is presented in Tables 3, 4, 5. The data extraction process identified two randomized controlled trials [26, 43], two prospective cohort studies [20, 42], and one retrospective investigation [28]. In total, 190 implants were examined: 91 provided with customized healing abutments and 99 with conventional healing abutments. The studies included implants placed in anterior teeth, premolars, and molars. Follow-up periods ranged from 6 to 36 months. The survival rates of the implants over the respective observation times were 100% in all groups.

**Table 3:** Detailed information extracted from studies included in this systematic review [part 1]

Article	Publication Year	Study type		Follow-up	Implant positions	Study population N = all participants	Mean age of study population	Drop outs after implant placement	Timing of implant placement	Implant system	Guided implant insertion	Gap-filling graft material
Amato et al. [20]	2022	Prospective	Cohort study (5-arms) (multicentric)	6 months	Molars	N = 264 conv.: n_1_ = 67 conv.: n_2_ = 64 conv.: n_3_ = 71 abutment: n_4 _= 33 cust.: n_5 _= 32 male/female: n.a	n.a	n.a	Immediate implant placement after tooth extraction	T3, Biomet 3i tapered implants (Zimmer Biomet Holdings, Warsaw, Indiana, USA) (diameter of 5.0 mm)	Orientation surgical template	n.a
Chokaree et al. [26]	2024	Prospective	Randomized controlled clinical trial (2-arms) (monocentric)	6 months	Anterior teeth, premolars	N = 12 conv.: n = 6 cust.: n = 6 6 female 6 male	59.0 ± 4.8 years	No drop out	Immediate implant placement after tooth extraction	CMI IS-III Active implant (Neobiotech Co., Seoul, Republic of Korea)	Surgical guide	Bone grafting material (no further information)
Crespi et al. [28]	2023	Retrospective	Cohort study (2-arms) (monocentric)	36 months	Premolars, molars	N = 50 conv.: n = 27 cust.: n = 23 31 female 19 male	55.7 ± 8.8 years	50 out of 87	Late implant in healed site	Outlink® implabts (Sweden & Martina, Padua, Italy) Perfect implants (Avenir, Santarcangelo di Romagna, Italy)	n.a	n.a
Hu et al. [42]	2018	Prospective	Cohort study (2-arms) (monocentric)	6 months	Premolars, molars	N = 27 conv.: n = 16 cust.: n = 12 13 male 14 female	39.3 ± 13.1 years	No drop out	Immediate implant placement after tooth extraction	BL implants (Straumann Group, Basel, Switzerland) (diameter of 4.1 or 4.8 mm)	n.a	Allogenic (deproteinized bovine bone mineral (DBBM) grafting BioOss, Geistlich Pharma AG, Wolhusen, Switzerland)
Perez et al. [43]	2020	Prospective	Randomized clinical trial (2-arms) (monocentric)	12 months	Anterior teeth, premolars	N = 36 conv.: n = 18 cust.: n = 18 17 female 19 male	54.9 ± 17.5 years	No drop out	Immediate implant placement after tooth extraction	BLT implants (Straumann Group, Basel, Switzerland)	n.a	Allogenic (alloplastic bone graft substitute, GUIDOR easy-graft CRYSTAL, Sunstar Suisse SA, Etoy, Switzerland)

**Table 4:** Detailed information extracted from studies included in this systematic review [part 2]

Article	Control group(s) CONVENTIONAL healing abutments	Test group CUSTOMIZED healing abutments	Material and fabrication of customized healing abutment	Prosthetic restoration	Outcome variables primary	Outcome variables secondary	Implant/prosthetic survival conventional	Implant/prosthetic survival customized	Randomization process
Amato et al. [20]	1) Healing abutment with a 5-mm–wide body (Standard One-Piece ITHA54, Biomet 3i) 2) healing abutment with a 6.0-mm–wide body (EP6 One-Piece ITHA64, Biomet 3i) 3) healing abutment with a 7.5-mm–wide body (EP7 One-Piece ITHA74, Biomet 3i) 4) provisional prosthesis (Titanium Temporary Cylinders, Zimmer Biomet) that were immediately placed out of occlusion	Customized healing abutment shaped from PreFormance Temporary Cylinders (Biomet 3i) in the shape of the inner size of the alveolus	Resin hand-made	Screw-retained crown (6 months after implant placement)	Volumetric tissue changes	*none*	100%	100%	Random selection (no further information)
Chokaree et al. [26]	Prefabricated titanium healing abutment	Customized PEEK healing abutment	PEEK CAD/CAM	Screw-retained, all ceramic crown (6 months after implant placement) (former: screw-retained provisional crown after 4 months of healing)	Pink esthetic score (PES) (PES-change); soft tissue analysis: –horizontal tissue linear alterations: mean buccal change (MBC) –mean total change (MTC) –vertical linear; tissue alterations: mesial papillae height variation (mPHv), distal papillae height variation (dPHv), papillae height variation (PHv), midfacial height variation (MFHv) hard tissue analysis: vertical bone loss mesial (mBC) and distal (dBC) volumetric analysis: –buccal volume variation (BV) –total volume variation (TV)	Pain score	100%	100%	Balanced randomization (ratio 1:1) (no further information)
Crespi et al. [28]	Standard healing abutment	Cross-linkable acrylic resin (according to principles of the Biologically Oriented Preparation Technique (BOPT))	Resin hand-made	Cement- or screw-retained crown (3 months after implant placement)	Changes in the soft tissue: –crestal width –Papilla Index –peri-implant Probing Depth (PPDs) –keratinized Mucosa Width (KMW) –bleeding on Probing (BOP) changes in width of alveolar ridge	Adverse events: dental implant failing criteria	100%	100%	No information/no possible (retrospective) linear analyses were performed by a blind collector
Hu et al. [42]	Fit titanium healing abutment (Straumann RC Healing Abutment, conical shape)	Circumference of a PEEK (polyether ether ketone) healing abutment (Straumann Regular CrossFit Healing Abutment, customizable, D 7 mm, polymer) was roughened by carving some grooves; light-polymerizing acrylic resin (ShoFu, Kyoto, Japan) was injected in the space between the abutment and the working cast to sculpt the emergence profile of the natural tooth	Resin hand-made	No information	Vertical bone changes horizontal bone changes, facial mucosal level (FML) (distance between mid-buccal mucosal margin to reference tooth cusps)	*none*	100%	100%	No clear information (all surgical sites were distributed into 2 groups according to the size of the alveolar socket (for sockets too large to be closed by titanium healing abutments))
Perez et al. [43]	Standard healing abutment (cylindrical/convex)	Fabricated in resin after an impression taken with the pick-up impression copings using a polyether material (insertion within 24 h after implant placement)	Resin rand-made	Screw-retained crown (4 months after implant placement)	Papilla Index Pink Esthetic Score (PES)	(a) Comparison of the changes of soft tissues facial soft tissue level, width of keratinized gingiva (b) comparison of changes of the peri-implant bone level (c) comparison of implant success rate (d) comparison of implant failure and complication rate (biological and technical complications)	100%	100%	Using a random permutation sequence generator (Statistics Toolbox, MatLab 7.11, The MathWorks, Natick, Massachusetts) without any kind of minimization of confounding factors such as age, smoking habit, gender, and tooth position statistician was blind clinicians were calibrated

**Table 5:** Detailed information extracted from studies included in this systematic review [part 3]

Article	Primary Outcome Outcome for peri-implant conditions and aesthetic results	Secondary outcome Outcome for pain score of clinical treatment
Amato et al. [20]	• Observed volumetric soft tissue changes appeared to vary based on the use of different healing abutment sizes • The use of a customized healing abutment resulted in preservation of the original horizontal dimension of the molar soft tissue • Best results were achieved using a customized healing abutment or a provisional restoration, in which the transmucosal area matched the inner size of the alveolus showing minimal dimensional tissue reduction • Study protocol that utilizes customized healing abutments can be used to promote optimal esthetic results in immediate replacement of single teeth in the molar area	*Not reported*
Chokaree et al. [26]	• Customized group exhibited a significantly lower PES change than the prefabricated group • No difference in the preservation of marginal bone	• Customized group exhibited a significantly lower pain score than the prefabricated group
Crespi et al. [28]	• Immediately loaded implants with customized healing abutments showed better results in terms of alveolar soft tissue thickness and width of the keratinized mucosa compared with those of conventional group • Side effects count appeared to be very similar between the two groups • Customized healing abutments led to significant augmentation of the alveolar width more than twice that registered in the conventional group	*Not reported*
Hu et al. [42]	• For immediate implants placed into posterior sockets, customized healing abutments can facilitate closure of large sockets • Despite more pronounced incomplete defect fill, healing abutments consisting of PEEK and resin did not render an increased risk for peri-implant bone loss or soft tissue recession during the early healing period • Differences in bone width decrease did not reach statistical significance between both groups	*Not reported*
Perez et al. [43]	• Customized healing abutment group showed the most favorable outcomes (in terms of Papilla Index and marginal bone loss) in case of immediate implant that received a peri-implant bone grafting procedure • Use of a treatment strategy, based on the site-specific characteristics of the post-extraction soft tissues and with a concave shape at the base, is potentially able to give better results in terms of both soft and hard tissues	*Not reported*

Four studies compared customized and conventional healing abutments following immediate implant placement after tooth extraction, sometimes with simultaneous bone grafting [20, 26, 42, 43]. One study focused on two approaches for late implant placement in healed sites [28]. The study protocols varied in the materials used for customized healing abutments. Four studies utilized hand-made resin healing abutments [20, 28, 42, 43]. While one study investigated healing abutments made from polyether ether ketone using a computer-aided design/computer-aided manufacturing (CAD/CAM) process [26]. Healing abutments of conventional groups were prefabricated stock abutments out of Titanium (Tables 3, 4, 5). Conventional healing abutments in the comparison groups were prefabricated stock abutments made of titanium (Tables 3, 4, 5). All studies underwent a risk-of-bias analysis, with all showing a low risk of bias (Table 6).

**Table 6:** Risk-of-bias analysis for the included studies

RoB2 tool						
Article	Overall bias	Randomization process	Deviations from intended intervention	Missing outcome data	Measurement of the outcome	Selection of the reported result
Chokaree et al. [26]	Low risk	Low risk	Low risk	Low risk	Low risk	Low risk
Perez et al. [43]	Low risk	Low risk	Low risk	Low risk	Low risk	Low risk

Joana Briggs Institute Critical Assessment questionnaires					
Author	Overall bias	Yes [%]/question number	No [%]/question number	Unclear [%]/question number	Not applicable [%]/question number
Amato et al. [20]	Low risk	72.7%/2–5, 7, 9–11	18.1%/1, 6	9.1%/8	0.0%/–
Crespi et al. [28]	Low risk	72.3%/1–5; 7, 8, 11	9.1%/10	18.2%/6, 9	0.0%/–
Hu et al. [42]	Low risk	81.8%/1–5, 7, 9, 10, 11	0,0%/x	18.2%/6, 8	0.0%/–

### Peri-implant soft and hard tissue outcomes, peri-implant tissue health status, and aesthetic results (primary outcome)

In terms of peri-implant soft tissue, two of the five studies reported superior outcomes with customized healing abutments. Specifically, this means that Amato et al. [20] found a significantly lower bucco-palatal volumetric reduction around the keratinized tissue (*p* < 0.001), and Perez et al. [43] reported significantly better results in papilla indices (*p* < 0.001) — each for customized healing abutments. The other studies confirmed stable keratinized tissue with similar values for both groups [20, 26, 42]. Additionally, Hu et al. [42] noted that customized healing abutments could facilitate the closure of large sockets for immediate implants, especially in posterior sockets.

Regarding hard tissue changes, the studies by Chokaree et al. [26], Hu et al. [42], and Perez et al. [43] showed no significant difference in vertical bone changes between the two groups over observation periods ranging from 6 to 12 months. The horizontal ridge dimensions with customized healing abutments remained almost stable over 6 months in the study by Amato et al. [20]. Crespi et al. [28] observed a significant increase in alveolar width from baseline (implant placement) to the 3-month follow-up in both groups (*p* < 0.001), with a significantly greater increase in the customized healing abutment group (+2.5 ± 0.5 mm) than in the conventional group (+1.0 ± 0.9 mm) (*p* < 0.001). During the observation period from 3 months to 3 years, alveolar widths remained unchanged in both groups [28].

In terms of peri-implant tissue health status, except bone vertical bone changes, Crespi et al. [28] investigated those aspects. However, they found no significant differences in “Peri-implant Probing Depth” or “Bleeding on Probing [28].

Regarding aesthetic outcomes, Chokaree et al. [26] and Perez et al. [43] assessed changes in the Pink Aesthetic Score (PES) and reported mixed findings. Chokaree et al. [26] found significantly better PES values in the customized healing abutment group after 6 months (*p* = 0.022), while Perez et al. [43] reported no significant difference in the PES after 12 months. Other parameters for aesthetic outcomes were not investigated.

### Patient-reported outcome measures (PROMs) of clinical treatment (secondary outcome)

Only Chokaree et al. [26] evaluated PROMs in form of the pain development during clinical treatment. Pain assessments were conducted at the time of definitive crown insertion (baseline), as well as 2 and 24 h afterward, using a numerical rating scale (NRS). The pain NRS score was significantly higher for patients with conventional healing abutments at the time of prosthesis insertion (*p* = 0.003) and 2 h later (*p* = 0.013). After 24 h, no significant differences were observed [26]. Further parameters of PROMs were not investigated in the other studies included.

## Discussion

This systematic review aimed to elucidate the benefits of customized healing abutments compared with conventional ones during the non-submerged healing process. The null hypotheses were rejected because advantageous effects were observed in biological and aesthetic parameters, as well as in PROMs in form of pain development.

The review was designed to conduct a comprehensive literature search across four well-known databases. To minimize the risk of missing relevant studies, synonymous search terms for customized and conventional healing abutments were carefully evaluated and included in the search strategy. Despite this thorough approach, the scientific evidence remains limited, with only two randomized controlled trials, two prospective cohort studies, and one retrospective cohort study meeting the inclusion criteria. A 2022 investigation by Crespi et al. [47] was excluded because it examined the same study group as a 2023 investigation by the same authors [28]. Given the follow-up durations, it should be noted that only short-term performance could be assessed; only Crespi et al. [28] provided 3-year data, while the other studies reported results between 6 and 12 months.

Overall, the included studies exhibited a low risk of bias. Two different analysis methods were used depending on the study design. For randomized clinical trials, the RoB2 tool was employed. Although this tool is detailed and comprehensive, it requires calibration exercises and intensive training for reliable use [40]. Nevertheless, it is well established in the field. For cohort studies, the Joanna Briggs Institute Critical Assessment questionnaire was utilized, which is an approved method for assessing methodological quality based on 11 questions [41].

Various parameters related to soft and hard peri-implant tissue and aesthetic aspects were observed over time, leading to the use of different methodologies and measurements (Tables 3, 4, 5). In order to extract as much information as possible from the existing literature, all objectifiable data that take into account hard and soft tissue changes as well as peri-implant health should finally be analyzed. The data were too heterogeneous to conduct a meaningful meta-analysis in addition to the systematic review, highlighting the need for more data collection in the future. Besides the scarcity of data upon the health of peri-implant tissue health, a trend toward better soft and hard tissue outcomes and aesthetic benefits emerged, although the conclusions were based on only five studies [20, 26, 28, 42, 43].

Lenz et al. [37] conducted a similar systematic review in 2023, which also suggested a favorable peri-implant response. However, the conclusions should be interpreted with caution. Lenz et al. [37] included studies comparing non-submerged customized healing abutments with standard submerged healing procedures using conventional abutments as control groups. The authors of the present study believe that direct comparisons using the same healing method reduce the risk of potential side effects and yield more meaningful outcomes. Furthermore, Lenz et al. [37] focused solely on immediate implant placement, whereas the present study also included data on late implant placement.

In their meta-analysis, Troiano et al. [10] found that implants placed using a non-submerged technique had a slightly higher rate (2%) of early implant failures (*p* = 0.008). Late implant failures after more than 6 months and marginal bone loss appeared similar between groups (*p* = 0.22). Comparing submerged healing with conventional healing abutments and non-submerged healing with customized healing abutments must be done cautiously because it may introduce a higher risks of bias. Nonetheless, many studies comparing these two treatment concepts reported similar or slightly better peri-implant soft and hard tissue outcomes with customized healing abutments [30–32, 48]. This is noteworthy because customized healing abutments might be considered to be at a disadvantage due to their association with non-submerged healing, which carries a slightly higher complication rate [10, 21].

Single-arm studies without control groups have also revealed positive peri-implant hard and soft tissue outcomes when customized healing abutments were used, regardless of material (e.g., flowable resin composite, acrylic resin, PMMA CAD/CAM fabricated, or polyether ether ketone CAD/CAM fabricated) [11, 29, 33–36]. In some of these studies, the observation periods extended up to 8 years, allowing for the assumption of high implant success rates, though these studies lacked comparison groups [33, 34]. Thus, available studies align with the favorable outcomes of customized healing abutments, showing stable peri-implant conditions without disadvantages. However, the influence of individual materials could not be assessed because of limited data.

Pain development during clinical treatment, a secondary outcome in the field of PROMs, was only examined in one of the five included studies, making an evidence-based statement limited for robust conclusions. Chokaree et al. [26] reported lower NRS scores during the first 2 h when using customized healing abutments, consistent with findings by Beretta et al. [25]. Both studies noted significant differences in pain scores at the time of crown placement (*p* < 0.001) and 2 h (*p* < 0.001). No differences were observed 24 h post-treatment (*p* = 0.30) [25]. However, these studies included only 6 and 10 patients per test group, respectively [25, 26].

Beyond research data, the perceived clinical success of customized healing abutments is grounded in sound implantological and prosthetic principles. For instance, prerequisites such as sufficient keratinized mucosa, primary stability, and correct implant positioning are crucial for establishing and maintaining stable soft tissue and bone conditions. Additionally, the correct design of the emergence profile according to the aesthetic biological contour concept for implant restorations is vital, often beginning at the early surgical stage [25, 26, 43].

The use of customized healing abutments may reduce the patient chair time during prosthesis insertion while still achieving an aesthetically pleasing result. With individually shaped peri-implant soft tissue, the need for stepwise displacement and compression of the gingiva is minimized or eliminated when the final restoration or abutment is placed [24]. Further studies investigating the potential for increased efficiency would be valuable. Additionally, the clinical treatment time might be reduced by using CAD/CAM-fabricated customized healing abutments, eliminating the time-consuming chairside fabrication of self- or light-curing resins. These CAD/CAM abutments can be sterilized, and outsourcing their preparation to a skilled dental technician could lower operating costs compared with those of a dental office. Moreover, involving the dental technician early in the treatment process could enhance the workflow [24]. Important considerations and decisions should always be discussed and resolved within the restorative team [3]. However, the digital approach in implantology and implant prosthetics helps streamline laboratory and clinical protocols while enhancing quality management through standardization [1].

Despite the small number of included studies and the relatively short follow-up periods, integrating customized healing abutments into the clinical workflow appears to be a valuable technique for achieving predictable outcomes in implantology and implant prosthetics. The use of anatomically designed abutments could be particularly advantageous, and their incorporation into clinicians’ (digital) workflows is recommended.

## Conclusion

Within the limitations of the present systematic review, it can be concluded that customized healing abutments

- support the peri-implant soft tissue during non-submerged healing and predictably shape it which can improve aesthetic outcomes,
- offer advantages regarding peri-implant soft and hard tissue results compared with conventional healing abutments, and
- can be recommend for immediate and late implant placement procedures.

Nevertheless, further studies based on larger sample sizes and longer follow-up periods are required to validate these findings for sufficient long-term outcomes.

## Data Availability

No datasets were generated or analysed during the current study.
